# Integrated Transcript and Metabolite Profiles Reveal That *Eb*CHI Plays an Important Role in Scutellarin Accumulation in *Erigeron breviscapus* Hairy Roots

**DOI:** 10.3389/fpls.2018.00789

**Published:** 2018-06-21

**Authors:** Ruibing Chen, Xianghui Chen, Tingting Zhu, Jianghua Liu, Xing Xiang, Jian Yu, Hexin Tan, Shouhong Gao, Qing Li, Yichao Fang, Wansheng Chen, Lei Zhang, Baokang Huang

**Affiliations:** ^1^Department of Pharmacognosy, School of Pharmacy, Second Military Medical University, Shanghai, China; ^2^Department of Pharmaceutical Botany, School of Pharmacy, Second Military Medical University, Shanghai, China; ^3^Development and Utilization Key Laboratory of Northeast Plant Materials, School of Traditional Chinese Materia Medica, Shenyang Pharmaceutical University, Shenyang, China; ^4^Department of Pharmacy, Changzheng Hospital, Second Military Medical University, Shanghai, China; ^5^School of Forestry, Southwest Forestry University, Kunming, China; ^6^College of Life Science, Fujian Agriculture and Forestry University, Fuzhou, China

**Keywords:** *Erigeron breviscapus*, correlation analysis, scutellarin, chalcone isomerase, hairy root culture

## Abstract

Scutellarin, a flavonoid 7-O-glucuronide, is an essential bioactive compound of *Erigeron breviscapus* (Vaniot) Hand.-Mazz. used for the treatment of cerebrovascular diseases. However, due to overexploitation and overuse, *E. breviscapus* is facing the problems of extinction and habitat degradation. In this study, a correlation analysis between the transcript and metabolite profiles of methyl jasmonate (MeJA)-treated *E. breviscapus* at different time points indicated that chalcone isomerase (*Eb*CHI) was the primary contributor to scutellarin accumulation during flavonoid biosynthesis. *Eb*CHI was then further characterized as a chalcone isomerase that efficiently converted chalcone to naringenin *in vitro*. Optimal parameters derived by comparing different culture conditions were successfully used to establish hairy root cultures of *E. breviscapus* with a maximum transformation rate of 60% in B5 medium. Furthermore, overexpression of *Eb*CHI significantly enhanced scutellarin accumulation in *E. breviscapus* hairy roots with a maximum content of 2.21 mg g^-1^ (dw), 10-fold higher than that of natural roots (0.21 mg g^-1^ dw). This study sheds new light on a method of effective gene-based metabolic engineering by accurate and appropriate strategies and provides a protocol for hairy root cultures that accumulate high levels of scutellarin, providing a promising prospect for relieving the overexploitation and unavailability of *E. breviscapus* in the future.

## Introduction

*Erigeron breviscapus* (Vaniot) Hand.-Mazz., an important traditional Chinese herbal medicine, is broadly used for the treatment of cerebrovascular diseases and diabetes (Shi et al., 1998; Zhu et al., 1999; Qian et al., 2011) and was officially listed in the Chinese Pharmacopeia in 1997. It is well known that the bioactive components of *E. breviscapus* are flavonoid 7-O-glucuronides, including scutellarin and a small amount of apigenin 7-O-glucuronide (Chu et al., 2005). Flavonoid 7-O-glucuronides are always found in plants within the order Lamiales, such as *Prunella vulgaris, Salvia splendens*, and *Scutellaria baicalensis* (Noguchi et al., 2009). Scutellarin, an unusual 6-hydroxy-substituted flavonoid 7-O-glucuronide is accumulated in only a few *Erigeron* species, including *E. breviscapus*. Unfortunately, this important medicinal plant, which is distributed in Yunnan Province in China, is endangered due to overexploitation (Yu and Chen, 2002). Therefore, it is urgent to alleviate the resource shortage of *E. breviscapus.*

**FIGURE 1:**
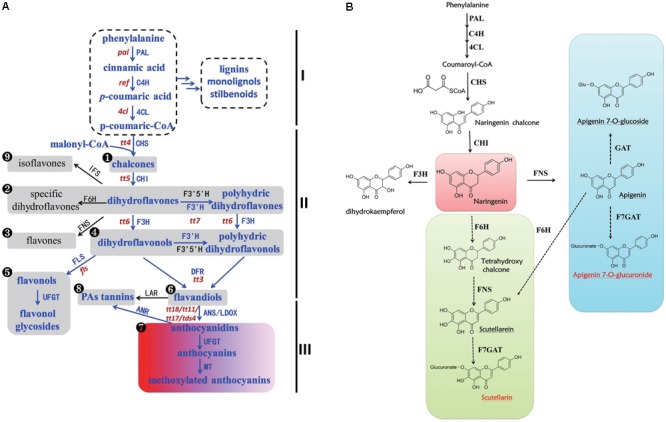
Biosynthesis of flavonoids in plants. **(A)** Biosynthesis of flavonoids in the plant kingdom. Part I is the phenylpropanoid pathway and its downstream metabolites. Part II is the flavonoid pathway, and part III is the anthocyanin pathway. The numbers in black circles indicate different types of flavonoid structure. The portion shown in blue is the pathway that exists in *Arabidopsis*, while the black portion exists in other plants. The lowercase words in red represent the mutants beside the corresponding genes in *Arabidopsis* (Bharti and Khurana, 2003; Petrussa et al., 2013). **(B)** Putative biosynthesis of scutellarin in *Erigeron breviscapus*. Full and dashed lines mark defined and putative steps in all species, respectively. The red words represent bioactive compounds in *E. breviscapus*, and the red square is a significant branch point. The green and blue squares are two different ways of producing scutellarin. Enzyme abbreviations are as follows: PAL, phenylalanine ammonia-lyase; C4H, cinnamate 4-hydroxylase; 4CL, 4-coumarate CoA ligase; CHS, chalcone synthase; CHI, chalcone isomerase; F3H, flavanone 3-hydroxylase; F3′H, flavonoid 3′-hydroxylase; F3′5′H, flavonoid 3′, 5′-hydroxylase; DFR, dihydroflavonol reductase; LDOX/ANS, leucoanthocyanidin dioxygenase/anthocyanidin synthase; UFGT, UDP-glucose: flavonoid 3-O-glucosyl transferase; MT, methyltransferase, ANR, anthocyanidin reductase; LAR, leucoanthocyanidin reductase; FLS, flavonol synthase, FNS, flavone synthase, F6H, flavonoid 6-hydroxylase; F7GAT, flavonoid 7-O-glucuronosyltransferase; GAT, glycosyl transferases; IFS, isoflavone synthase.

In general, flavonoid metabolites are sub-classified into several families including flavones, flavonols, flavandiols, flavan-3-ols, anthocyanidins, proanthocyanidins (PAs), flavanones, dihydroflavones, dihydroflavonols, and isoflavones according to their structures and the modifications to the A, B, and C rings known as the flavonoid backbone (Debeaujon et al., 2001; Shi and Xie, 2014; Takayuki et al., 2017). The biosynthesis of flavones and other flavonoids has been well characterized (**Figure 1A**) (Petrussa et al., 2013). Compared with the common flavonoid biosynthetic pathway, we hypothesized a potential biosynthetic pathway for scutellarin in *E. breviscapus* (**Figure 1B**) based on previous studies (Jiang et al., 2014; Chen et al., 2015b). On the one hand, naringenin was transformed into apigenin by flavone synthase (FNS), and then, apigenin could be converted to apigenin 7-O-glucoside and apigenin 7-O-glucuronide through glycosylation. On the other hand, scutellarein was produced through hydroxylation by flavonoid 6-hydroxylase (F6H) and subsequent oxidation by FNS from naringenin; scutellarein could then be converted to scutellarin by glycosylation (UDP-glucose: flavonoid 7-O-glucosyl transferase, F7GAT). However, we still do not know which pathway contributes the most to the accumulation of scutellarin in *E. breviscapus.*

Because of the rapid development of metabolomics and transcriptome research techniques, the principle of transcript and metabolite co-occurrence, particularly gene-metabolite network analysis, has become a powerful tool for decoding the function of genes and screening rate-limiting enzymes (Saito and Matsuda, 2010). The plant hormone methyl jasmonate (MeJA) has broadly been used to induce secondary metabolism and make changes to transcripts and metabolites (De Geyter et al., 2012). To make a gene-metabolite network, the canonical correlation analysis method using the Pearson correlation coefficient requires information about both gene expression and metabolic changes (Cline et al., 2007). Combining transcripts and metabolites, this strategy was successfully used to establish gene-metabolite networks of MeJA-mediated regulation of secondary metabolism for a rate-limiting enzyme screening of lignan biosynthesis in *Isatis indigotica* (Chen et al., 2015a) and the rosmarinic acid biosynthetic pathways in *Salvia miltiorrhiza* (Xiao et al., 2010). However, the effect of MeJA on the biosynthesis of scutellarin in *E. breviscapus* still remains to be elucidated.

The hairy root, induced by a soil bacterium, *Agrobacterium rhizogenes*, is widely used in biotechnological applications to replace natural sources for the high production of valuable secondary metabolites (Chilton et al., 1982; Mishra and Ranjan, 2008; Sheela and Ramesh, 2011), such as high accumulation of the anticancer compound plumbagin in *Plumbago rosea* hairy root (Jose et al., 2016) and p-hydroxybenzoic acid (pHBA) in *Beta vulgaris* hairy root (Rahman et al., 2009). The hairy root system also shows high stability and fast, hormone-independent growth. Based on an understanding of flavonoid biosynthesis, these advantages of the hairy root system make it a good prospect for solving the resource shortage of *E. breviscapus*.

Here, a gene-to-metabolite strategy revealed the MeJA-specific responses of scutellarin biosynthesis in *E. breviscapus*. We demonstrated that the abiotic elicitor MeJA dramatically induced the biosynthesis of scutellarin in *E. breviscapus*. Furthermore, through correlation analysis, a gene-metabolite network of scutellarin biosynthesis was constructed, which demonstrated that *Eb*CHI is a rate-limiting enzyme for the biosynthesis of scutellarin. Using the hairy root system for metabolic engineering, the content of scutellarin was dramatically increased: approximately 10-fold higher in *Eb*CHI-overexpressing hairy roots than in wild-type hairy roots.

## Materials and Methods

### Plant Materials

*Erigeron breviscapus* plants were grown in a glasshouse at 22°C under 12 h of light at the Second Military Medical University, Shanghai, China. Two groups of 90-day-old plants were randomly selected and treated with 1.5 mM (MeJA, dissolved in 0.1% acetone) or 0.1% acetone (negative control). After treatment, the plants were harvested at distinct time points for RNA isolation (0, 1, 3, 6, 12, 24, and 36 h) and chemical analysis (0, 1, 2, 3, 4, 5, 6, 7, and 8 days). All time points contained three independent plants as biological repetitions.

### Reagents and Standards

Methyl jasmonate (95%), apigenin 7-O-glucuronide, scutellarin, apigenin, scutellarein, apigenin 7-O-glucoside, naringenin chalcone, naringenin (98%), and acetosyringone were all purchased from Sigma (United States). The antibiotics cefotaxime and hygromycin B were purchased from Sangon (China). The plant hormones 1-naphthaleneacetic acid (NAA) and 6-benzylaminopurine (6-BA) were purchased from Phytechnology Laboratories (United States). Ammonium acetate was purchased from Thermo Fisher Scientific (United States) and methanoic acid from Aladdin (China). Methanol and acetonitrile of high performance liquid chromatography (HPLC) grade were from Merck Company (Germany). A Milli-Q Reagent Water System was used to produce ultrapure water (Millipore, United States).

### RNA Isolation and Quantitative Reverse Transcription PCR Analysis

To assess the response of the flavonoid pathway to MeJA, quantitative reverse transcription PCR (qPCR) was used to analyze the gene transcripts at different time points after MeJA treatment. Total RNA was extracted using TRIzol Reagent (Tiangen, China) according to the manufacturer’s instructions. The quality and concentration of RNA were examined by agarose gel electrophoresis and a NanoDrop 2000 (Thermo Fisher Scientific, United States). A sample of 1 μg RNA was used for reverse transcription by First-Strand cDNA Synthesis SuperMix Reagent (Transgen Biotech, China).

The primers used for real-time quantitative reverse transcription PCR (qPCR) are listed in **Supplementary Table S1**. The *ubiquitin C* gene (*ubc*) was amplified as a housekeeping gene according to a previous study (Chen et al., 2015b). The qPCR experiment was performed in line with the manufacturer’s instructions (Takara, Japan) under the following conditions: 30 s pre-denaturation at 95°C, one cycle; 10 s denaturation at 95°C, 10 s annealing at 58°C, 20 s fluorescence collection at 72°C, and 40 cycles. The products of the qPCR were detected by agarose gel electrophoresis, which showed equally sized bands, as predicted. The comparative *C*_t_ method was used to analyze the gene expression. Each reaction was performed in triplicate. In comparison with the transcript levels of the untreated plants or root tissue, the relative expression levels were calculated.

### Metabolite Analysis

To determine the responses of flavonoid metabolites to MeJA in *E. breviscapus*, the metabolites were extracted and analyzed after MeJA treatment at different time points. Plants were dried at 50°C in an oven until they reached a constant dry weight (DW). The dried plant samples (2.0 mg) were ground into fine powder and extracted twice with 1 mL of 70% methanol under sonication for 30 min. The solutions were then merged and centrifuged at 15000 rpm for 30 min. The supernatant was diluted with 70% methanol to 2 ml. Before analysis, the extract solution was filtered through a 0.2 μm organic membrane (Woongki Science, Korea).

All samples were analyzed on an Agilent 1200 series HPLC interfaced to an Agilent 6410 triple-quadrupole mass spectrometer equipped with an electrospray ionization (ESI) source (Agilent Corporation, United States). A Zorbax SB-C18 column (3.5 μm, 2.1 × 150 mm, I.D. Agilent Corporation, United States) and a C18 guard column (5 μm, 4.0 × 2.0 mm, Agilent Corporation) were used to separate the compounds. A mobile phase consisting of acetonitrile and 5 mM ammonium acetate solution (the concentration of acetonitrile remaining at 30% for 2.0 min, v/v) was used at a flow rate of 0.3 ml min^-1^. The column temperature was maintained at 30°C. The injection volume was 10 μL. The ESI source in negative mode was chosen for five compounds. To obtain the richest relative abundance of precursor ions and product ions, the best parameters were chosen, and they are listed in **Supplementary Figure S1A**. **Supplementary Figure S1B** shows the full scan product ion spectra of the precursor ions of the five analytes. The MRM chromatograms of five standard compounds are shown in **Supplementary Figure S1C**. The standard sample concentration was 1 μg ml^-1^, and the injection volume was 10 μl for all samples. An untreated plant (day one) sample was used as a reference to calculate the relative content.

### Statistical Analysis

To find the rate-limiting step of the scutellarin metabolic pathway, correlations between the identified biosynthetic genes and five compounds were calculated by the Pearson correlation coefficient using *R*, based on the principle of co-occurrence between mRNA and metabolite levels (Saito and Matsuda, 2010; Xiao et al., 2015).

All experiments (hairy root clones, qPCR, and HPLC analysis) were performed with three independent replications. The standard errors were based on the biological triplicates.

The statistical significance of the metabolite and expression differences were analyzed by Student’s *t*-test, and the errors of different samples were used in a one-way analysis of variance (ANOVA) using SPSS 11.5 software (SPSS, Inc.). Transcripts from untreated plants were used as references at each time point to analyze the statistical significance of the changes in MeJA-treated plants, and the metabolite content of WT was used as a reference to analyze the statistical significance of the changes in *Eb*CHI-overexpressing hairy roots.

### Isolation and Characterization of *Eb*CHI

The full-length cDNA of *Eb*CHI (JF694272.1) was amplified by polymerase chain reaction, subcloned into the *p*Blunt-Zero vector according to the sequences published on National Center for Biotechnology Information (NCBI) (Transgene, China) and transformed into the *E. coli.* Trans1-T1 cell line (Transgene, China), which was employed for sequencing. All primers used in this study are listed in **Supplementary Table S1**.

The deduced polypeptide sequence of *Eb*CHI was aligned with those of *Cm*CHI (AEP37358.1), *Ct*CHI (ALG75881.1), *At*CHI (AEE79342.1), *Nt*CHI (NP_001312216.1), *Zm*CHI (NP_001149585.1), and *Ls*CHI (BAS69315.1) by MEGA v5.05.^1^ A phylogenetic tree was also constructed using the neighbor-joining method implemented in MEGA. The chalcone isomerase domain of *Eb*CHI was analyzed on NCBI.^2^ Secondary structure prediction was performed with SOPMA.^3^ The 3-D modeling and homology-based structural modeling were performed by Phyre^2^ online.^4^

To experimentally verify the subcellular localization of *Eb*CHI, the full-length *Eb*CHI coding sequence was fused in-frame to the *N*-terminus of enhanced green fluorescent protein (GFP) through the NcoI and BglII restriction sites of the *p*1301-GFP vector. Using polyethylene-glycol-mediated transformation, the vectors *p*1301-*Eb*CHI-GFP and *p*1301-GFP were individually introduced into protoplasts isolated from 14-day-old etiolated rice stems (Zhang et al., 2011; Tan et al., 2012), which were grown in a dark room on sucrose-containing 1/2 MS agar medium after sprouting from seeds in continuous light (You et al., 2013). The transient expression of GFP was visualized using a confocal laser scanning microscope (Nikon, Japan) as described by Tan et al. (2015).

### The Activity of *Eb*CHI *in Vitro*

To clone *Eb*CHI into the expression vector *p*ET-32a, a pair of primers, *p*ET-32a-*Eb*CHI-F and -R, were designed (**Supplementary Table S1**). The PCR product was cloned into *p*ET-32a through the BamHI and EcoRI restriction sites to express a recombinant 6 × His-*Eb*CHI protein. After confirmation by sequencing (GENEWIZ, China), the recombinant plasmid and empty vector pET-32a were separately transformed into the *E. coli.* cell line BL21 (DE3) (TransGen Biotech, China) by the heat shock method. The transformed cells were used to inoculate 200 mL of LB medium containing ampicillin (75 μg mL^-1^), grown at 37°C to an OD600 of 0.6, and then induced with 1.0 mM Isopropyl β-D-thiogalactoside (IPTG) at 16°C for 12 h. The His-tagged *Eb*CHI protein was purified at 4°C using nickel-chelate affinity chromatography according to the manufacturer’s instructions (Bio-Rad Laboratories, United States). The protein concentration was determined using bovine serum albumin (BSA) as a standard and visualized on 12% acrylamide gel stained with Coomassie Blue. Western blotting verified that the recombinant proteins had a His-tag in the *N*-terminus. The proteins were detected with the anti-rabbit mAb His antibody (Cell Signaling Technology, United States), and a secondary antibody (anti-rabbit IgG, horseradish peroxidase (HRP)-linked antibody).

For the *in vitro* enzyme assay, the reaction mixture contained: 85 μL Tris-HCl buffer (50 mM, pH 7.5), 5 μL purified recombinant *Eb*CHI protein (0.3 μg μL^-1^), and 10 μl naringenin chalcone (1.0 mM) dissolved in methanol (Qin et al., 2011). The enzyme assay was incubated at 30°C for 5 min and then extracted with 250 μL ethyl acetate. After removing the ethyl acetate, the residue was dissolved in 100 μL methanol and analyzed by HPLC on an Agilent Zorbax SB-C18 column (5 μm, 4.6 × 250 mm; Agilent, United States), eluted with a 33% (v/v) acetonitrile phase with 0.1% (v/v) methanoic acid in water at a flow rate of 1.0 ml min^-1^ at 25°C, and monitored at 254 nm.

### Plant Transformation and Hairy Root Culture

The hairy root, induced by *A. rhizogenes*, is widely used in biotechnological applications to replace natural sources for the high production of valuable secondary metabolites. Based on an understanding of flavonoid biosynthesis, these advantages of the hairy root system make it a good prospect for solving the resource shortage of *E. breviscapus*. Therefore, optimal parameters for establishing hairy root cultures of *E. breviscapus* were investigated.

To obtain sterile seedlings, the harvested *E. breviscapus* seeds were pre-treated with 75% alcohol for 1 min and washed three times with 60 mL distilled water. Then, the sterilized seeds were incubated between several layers of sterilized wet filter paper and cultured on MS solid medium for germination. The seedlings were grown at 25°C under a 12 h light and 12 h dark photoperiod cycle.

To induce hairy roots with the disarmed *A. tumefaciens* strain C58C1 (pRiA4) under optimal conditions, sterile leaf sections were pre-cultured on solid medium (MS, 1/2 MS, B5, and 1/2 B5 were compared) supplemented with 1 mg L^-1^ 6-BA, 1 mg L^-1^ NAA, 30% sucrose and 0.8% agar (pH 5.8) for 2 days. Then, the pre-cultured explants were submerged in a bacterial suspension (OD600 = 0.4, 0.6, and 0.8 were compared) supplemented with 100 μM acetosyringone for several minutes (5, 10, and 15 min were compared), blot-dried on sterile filter paper and then placed on the corresponding solid medium (MS, 1/2 MS, B5, or 1/2 B5) supplemented with 30% sucrose and 0.8% agar (pH 5.8) at 25°C in darkness for several days of co-cultivation (0, 1, 2, and 3 days were compared).

After co-cultivation, the induced explants were washed with 60 mL sterilized water three times and blot-dried on sterile filter paper, then transferred to the corresponding solid medium supplemented with 30% sucrose, 0.8% agar (pH 5.8), 500 mg L^-1^ cefotaxime and 10 mg mL^-1^ hygromycin in darkness. After 2 weeks, hairy roots were derived from the sterile leaves. The hairy roots that exceeded 2 cm were excised and cultivated individually on the corresponding solid medium supplemented with 30% sucrose, 0.8% agar (pH 5.8), 10 mg mL^-1^ hygromycin and different concentrations of cefotaxime for 4 weeks (the concentration of cefotaxime changed from 300 to 100 mg mL^-1^ after 2 weeks). Rapidly growing root lines that showed hygromycin resistance with no bacterial contamination were used to establish hairy root lines in 250-mL Erlenmeyer flasks containing 200 mL of the corresponding liquid medium supplemented with 30% sucrose and 10 mg mL^-1^ hygromycin on an orbital shaker at 100 rpm in darkness at 25°C.

The fresh weight (FW) of root tissues from the culture flasks (determined as the difference in flask weight with and without the harvested root tissues) was recorded every week after inoculation, and the information was used to draw growth rate curves. At week 10, the hairy roots were harvested for DNA and RNA extraction and metabolite analysis.

### Generation and Analyzes of *Eb*CHI-Overexpressing Hairy Roots

To construct the *Eb*CHI overexpression vector, the full-length *Eb*CHI cDNA without a stop codon was cloned into the plasmid *p*HB-flag using the BamHI and SpeI restriction sites to generate the *p*HB-*Eb*CHI-flag construct. The primers *p*HB-*Eb*CHI-F and *p*HB-*Eb*CHI-R were used and are listed in **Supplementary Table S1**. The plasmids *p*HB-*Eb*CHI-flag and *p*HB-flag were separately transformed into the *A. tumefaciens* strain C58C1 by the heat shock method. Using the plant transformation method mentioned above, *p*HB-*Eb*CHI-flag (*Eb*CHI-OVX), pHB-flag (CK) introduced and non-plasmid-introduced (WT) *A. tumefaciens* were separately introduced into *E. breviscapus* to generate transgenic hairy root cultures. At week 10, the hairy roots were harvested for DNA extraction and RNA extraction. Genomic DNA was isolated and then used in a PCR analysis for the detection of the presence of the exogenous *Eb*CHI. The primer *p*HB-*Eb*CHI-1F and Rbcs-R were specifically designed to cover the 3′ terminus of the *Eb*CHI sequence and the vector sequence (*Eb*CHI+vector: 243 bp + 506 bp) for detecting exogenous *Eb*CHI transformations. Primers for the *rolB* gene in the *A. tumefaciens* genomic DNA and the hygromycin resistance gene (*hyg*) in the plasmid *p*HB-flag were also used together to make sure that the transformations were successful.

## Results

### Gene Expression Profiles and Scutellarin Accumulation in Different Tissues

**FIGURE 2:**
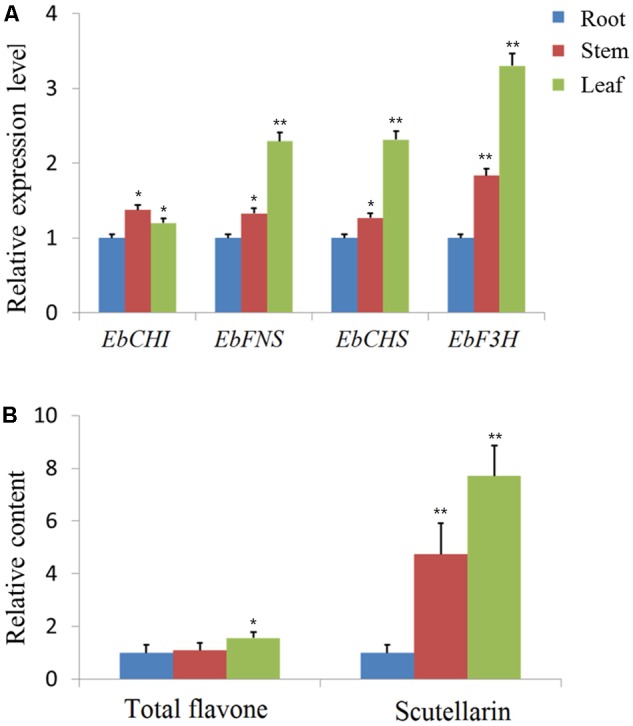
qPCR analysis of genes and content analysis of scutellarin in different organs. **(A)** Using the 2^-ΔΔCt^ method, the transcript abundance of each gene was normalized to *ubiquitin C* (*ubc*) as a housekeeping gene and compared with that in the root as a reference to calculate the relative expression level. **(B)** The contents of total flavone and scutellarin in different organs. The content of the root tissues was used as a reference to normalize and calculate the relative contents. The level of significance obtained with a Student’s *t*-test is indicated by the following: ^∗^, *p* < 0.05; ^∗∗^, *p* < 0.01.

Since the expression profile of a gene is often correlated with its function, the relative expression of the four genes *Eb*CHS, *Eb*CHI, *Eb*FNS, and *Eb*F3H was quantified in the total RNA isolated from different organs (root, stem, and leaf) through qPCR using gene-specific primers (**Supplementary Table S1**). The relative expression levels are shown in **Figure 2A**, which indicated that all genes were highly expressed in leaves except for the similar expression of *Eb*CHI in the three tissues. In addition, the contents of total flavones and scutellarin were both highest in leaves and lowest in roots. The content of scutellarin was 7.7-fold higher in leaves than in roots (**Figure 2B**).

### MeJA-Induced Changes in the Transcript Profiles

**FIGURE 3:**
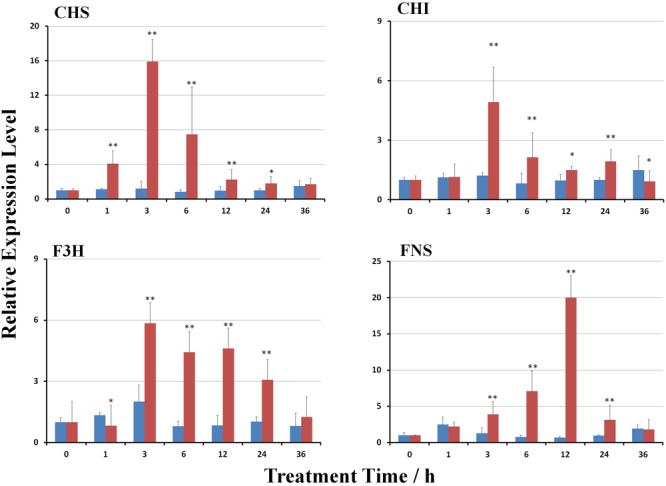
Effect of MeJA on gene transcripts in the flavonoid biosynthetic pathway. The untreated control is shown in blue, and the MeJA-treated is in red. The gene transcripts at hour 0 after MeJA treatment were used as a reference to calculate the relative expression level. Significant differences between the treated and untreated plants at each time point were calculated with a Student’s *t*-test, as shown by the following: ^∗^, *p* < 0.05; ^∗∗^, *p* < 0.01.

MeJA treatment significantly induced the expression of genes involved in the biosynthesis of scutellarin (*p* < 0.01). The transcript levels of *Eb*CHS*, Eb*CHI*, Eb*F3H, and *Eb*FNS were gradually stimulated (**Figure 3**). The expression of *Eb*CHI, *Eb*CHS, and *Eb*F3H reached the highest levels at 3 h after treatment, but that of *Eb*FNS was at 12 h after treatment, followed by a reduction. The maximum mRNA transcript levels of *Eb*CHS*, Eb*CHI*, Eb*F3H, and *Eb*FNS were 15.9-, 4.9-, 5.8-, and 19.9-fold higher than those of the control (untreated plants), respectively.

### MeJA-Induced Changes in the Metabolic Accumulation Profiles

To examine the response of metabolites to MeJA treatment, a liquid chromatography – tandem mass spectrometry (LC-MS/MS) method was established to determine the levels of five metabolites involved in scutellarin biosynthesis in *E. breviscapus*, including scutellarin, apigenin 7-O-glucuronide, apigenin, scutellarein, and apigenin 7-O-glucoside (**Supplementary Figure S1**).

**FIGURE 4:**
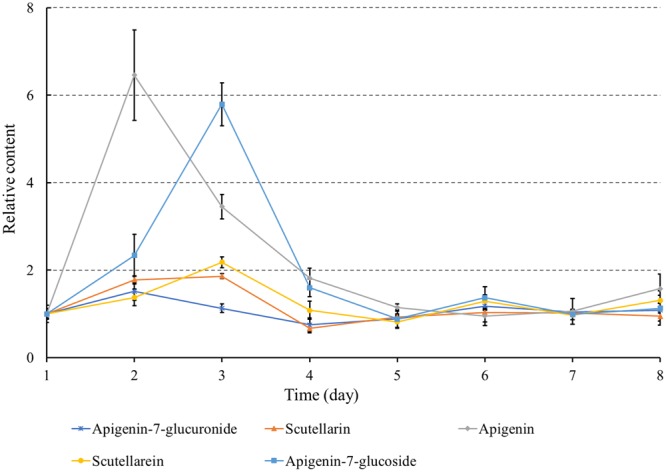
Stimulation of scutellarin biosynthesis in *E. breviscapus* by MeJA. Three independent biological samples were analyzed at each time point. The contents of five metabolites at day one after MeJA treatment was used as a reference to calculate the relative contents.

As shown in **Figure 4**, the relative contents of these five compounds were induced to varying degrees. The content of scutellarin was increased by 1.86-fold by MeJA treatment. Apigenin was increased more than six-fold in MeJA-induced cultures compared with control. As shown in **Figure 1B**, apigenin, as a common precursor, could be separated into two different pathways to produce apigenin 7-O-glucuronide and scutellarin. The content of apigenin reached its highest level at day two, and most apigenin was converted to apigenin-7-glucoside at day three, not scutellarin. The contents of apigenin-7-glucuronide, scutellarein, and apigenin-7-glucoside were most increased, by 1.5, 2.1, and 5.4, respectively. The absolute DW contents are listed in **Supplementary Table S2**, which shows a higher content of the two major active compounds (scutellarin and apigenin 7-O-glucuronide) than of the other three.

### Integrated Gene Transcript and Metabolite Accumulation Analysis

**FIGURE 5:**
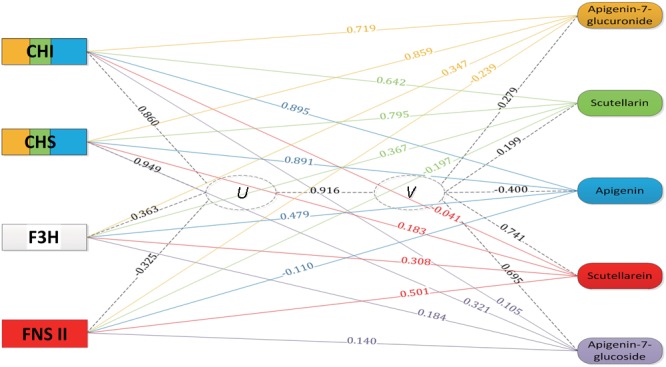
Gene-to-metabolite network in MeJA-induced *E. breviscapus* plants. Genes are represented by squares on the left and metabolites on the right. The canonical correlation coefficient between the canonical correlation variables (*U* and *V*) was 0.916. The correlation coefficients between the raw variables (genes and metabolites) and canonical correlation variables (*U* and *V*) are shown on the corresponding dotted line. The color in the gene square represents the level of gene-to-metabolite correlation, and edges were drawn when the variable correlation coefficient was >0.50; for example, a larger blue area represents more correlation to apigenin.

The accumulation profiles of the five compounds were combined with the expression profiles of four genes involved in the scutellarin biosynthesis pathway for an integrated analysis. To explore the correlation structure of the data, a canonical correlation analysis was performed, as shown in **Figure 5**. The first pair of canonical correlation variables (*U* and *V*) revealed a clear correlation between gene transcripts and the target metabolites: the canonical correlation coefficient was 0.916. Details of the correlation coefficients between the raw variables (gene or metabolite) and the canonical correlation variables (*U* or *V*) are listed in **Supplementary Table S3**. Variable correlation coefficient cutoff values (0.5 in this study) were applied to draw the edges as part of the exploratory study of gene-to-metabolite correlation structure. For example, the variable correlation coefficients between the *Eb*CHI transcript and the five compound accumulations were 0.719, 0.642, 0.895, -0.041, and 0.105, respectively, which indicated that *Eb*CHI, as an upstream gene, was correlated with apigenin 7-O-glucuronide, scutellarin and apigenin, and most closely with apigenin, but was not obviously correlated with scutellarein or apigenin 7-O-glucoside. In conclusion, the observations were as follows:

(1) two upstream genes (*Eb*CHI and *Eb*CHS) both showed strong correlations with scutellarin, apigenin 7-O-glucuronide and apigenin;
(2) *Eb*F3H, as a alternative pathway gene, showed very little correlation with any compounds involved in the biosynthesis of scutellarin;
(3) *Eb*FNS showed a correlation with only the accumulation of scutellarein.

According to the results of the integrated analysis, the upstream enzyme *Eb*CHI is a promising candidate for metabolic engineering to increase the content of the bioactive metabolite scutellarin.

### Characterization of *Eb*CHI

The cDNA of *Eb*CHI contains an ORF of 714 nucleotides corresponding to a 238-amino-acid protein with a calculated molecular mass of 25.64 kDa and a *p*I of 4.99 (**Supplementary Table S4**). Using the neighbor-joining method, a phylogenetic analysis was performed. The phylogenetic tree indicated that *Eb*CHI was clustered with other CHIs from the Compositae (**Supplementary Figure S2A**). *Eb*CHI had high homology to orthologues in other plants, which showed high conservation of the CHI enzyme during its evolutionary process (**Supplementary Figure S2B**). Like the CHIs in other plants, *Eb*CHI had only one chalcone isomerase domain (**Supplementary Figure S2C**). The secondary structure estimation showed that the *Eb*CHI peptide contained 36.55% alpha helices, 21.43% extended strands, 12.18% beta turns and 29.83% random coils. Alpha helices and random coils were the most abundant structural elements, penetrating through most of the *Eb*CHI secondary structure, while extended strands and beta turns were intermittently distributed in the protein (**Supplementary Figure S2D**). The 3-D structure of *Eb*CHI was predicted by Phyre^2^ using sequence-homology-based structural modeling^5^ (**Supplementary Figure S2E**). As shown in **Supplementary Figure S2F**, similar to that of GFP alone (**Supplementary Figure S2F-e**), the fluorescence of the fusion protein *Eb*CHI-GFP was distributed in the cytoplasm (**Supplementary Figure S2F-a**), which indicated that the conversion of chalcone to naringenin by *Eb*CHI should take place in the cytoplasm, in accordance with previous reports (Grotewold, 2001; Agati et al., 2012).

### The Activity of *Eb*CHI *in Vitro*

**FIGURE 6:**
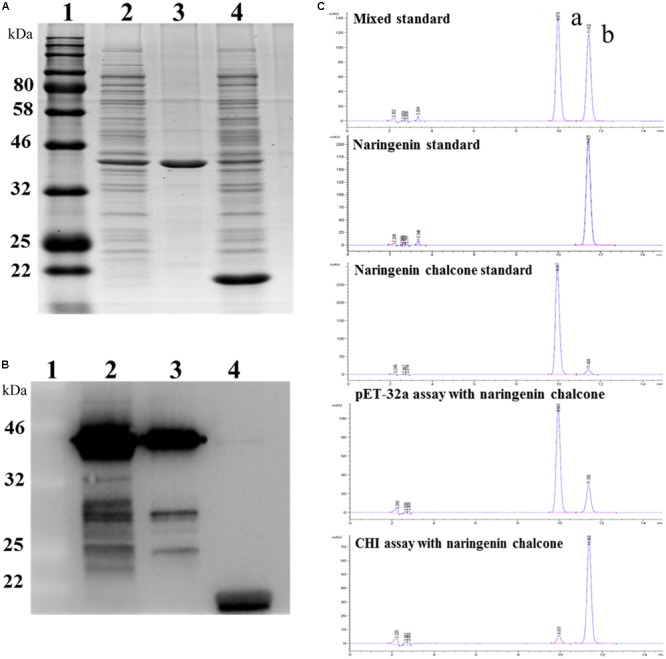
Functional characterization of *Eb*CHI *in vitro*. **(A)** SDS-PAGE analyzes of *Eb*CHI purified by Ni-NTA conjugation. Lane 1, protein molecular weight marker; lane 2, supernatant of *p*ET-32a-*Eb*CHI cell lysate; lane 3, purified *Eb*CHI protein; lane 4, crude enzyme extract from *p*ET-32a cell lysate. **(B)** Western blotting analyzes of *Eb*CHI. Lane 1, protein molecular weight marker; lane 2, supernatant of pET-32a-*Eb*CHI cell lysate; lane 3, purified *Eb*CHI protein; lane 4, crude enzyme extracted from pET-32a cell lysate. **(C)** Representative HPLC profiles of incubation of naringenin chalcone with purified *Eb*CHI protein or pET-32a protein. Peak a: naringenin chalcone; b: naringenin.

To test the function of the *Eb*CHI protein, a pET-32a-*Eb*CHI plasmid and an empty vector, pET-32a (control), were separately introduced into the *E. coli.* cell line BL21 (DE3). Upon induction by IPTG, a fusion protein, *Eb*CHI-His, was represented as the main soluble protein product (**Figure 6A**, lane 2). The purified *Eb*CHI-His protein presented a single distinct band (**Figure 6A**, lane 3). **Figure 6A**, lane 4, contained the crude enzyme extract from *E. coli* with the pET-32a vector. Western blotting of the purified recombinant *Eb*CHI-His protein confirmed its immune reactivity to anti-His antibodies (**Figure 6B**, lines 2 and 3). We used naringenin chalcone as a substrate to test its enzyme activity. The reaction products of recombinant *Eb*CHI-His were detected by HPLC monitored at 254 nm and identified by comparison to naringenin and naringenin chalcone standards (**Figure 6C**). On the one hand, the HPLC elution profiles of reaction products showed that *Eb*CHI yielded naringenin. On the other hand, the control produced a very low abundance of spontaneous product and left a majority of residual substrate. As predicted, *Eb*CHI converted naringenin chalcone into naringenin.

### Establishment of *E. breviscapus* Hairy Root Cultures

**FIGURE 7:**
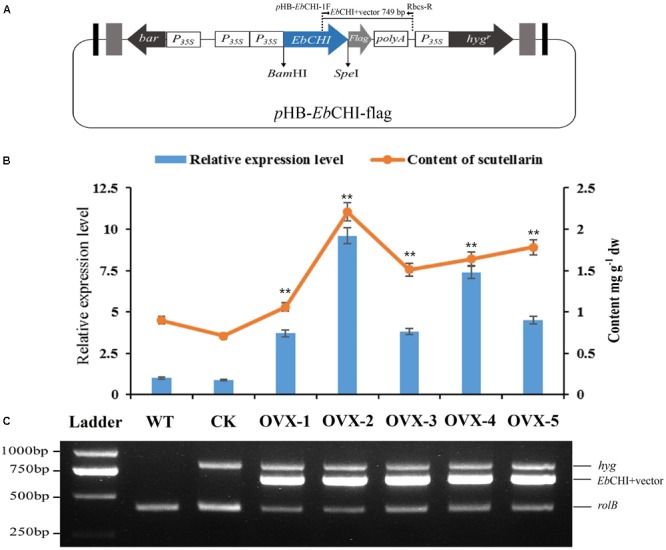
Hairy root cultures of *Eb*CHI-OVX. **(A)** Schematic representation of the overexpression vector used for hairy root establishment, *Eb*CHI-OVX. *P_*35S*_*, CaMV 35S promoter; *hyg^r^*, hygromycin resistance gene; *bar*, herbicide resistance gene; *Eb*CHI, *Eb*CHI coding sequence; *polyA*, poly(A) tail; *Flag*, flag-tag coding gene. **(B)** *EbCHI* transcript abundance and scutellarin accumulation in hairy root cultures. The level of significance obtained with a Student’s *t*-test is shown by the following: ^∗∗^, *p* < 0.01. **(C)** Analyzes of hairy roots at the molecular level. Ladder: DNA marker, WT: the hairy roots induced by blank C58C1 strain, CK: the hairy roots induced by *p*HB-flag, OVX-1∼5: the hairy roots induced by *p*HB-*Eb*CHI-flag; *rolB, hyg*, and *Eb*CHI indicate PCR fragments from the genomic DNA of hairy roots.

**FIGURE 8:**
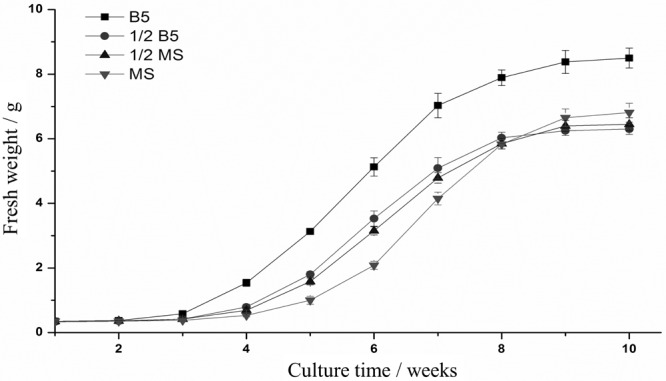
Growth rate of hairy roots in different mediums. The fresh weight (FW) of the root tissue was recorded every week.

In this research, the hairy root culture of *E. breviscapus* was first established. Based on the results of preliminary experiments, sterile leaf sections were optimally provided as donor explants for genetic transformation with the disarmed *A. tumefaciens* strain C58C1 (pRiA4). Successful transformation was indicated by the submergence of wounded leaves in the bacterial suspension (OD600 = 0.4, 0.6, and 0.8) for 5, 10, and 15 min. The optimal transformation rate (reaching a maximum of 60%) was observed when explants were induced in the bacterial suspension (OD600 = 0.6) for 10 min and co-cultured in B5 medium supplemented with 125 μM acetosyringone for 2 days (**Supplementary Table S5**). Established *E. breviscapus* hairy root cultures exhibited the typical morphological characteristics, with vigorous growth on phytohormone-free medium, extensive lateral branching and lack of geotropism (**Supplementary Figure S3**). In addition to these morphological characteristics, the *rolB* gene (located in the Ri T-DNA segment) was amplified from the genome of hairy roots, which confirmed the transformation of the hairy root cultures at the molecular level (**Figure 7C**). As shown in **Figure 8**, the growth rates varied in different media. The growth rate of hairy root in B5 medium was significantly higher than that in the other media (1/2 B5, 1/2 MS and MS). Meanwhile, the highest yield was also produced in B5 medium within 10 weeks.

### Overexpression of *Eb*CHI in *E. breviscapus* Hairy Root

The full-length *Eb*CHI cDNA without a stop codon was successfully subcloned into the plasmid *p*HB-flag using the BamHI and SpeI restriction sites to generate the *p*HB-*Eb*CHI-flag construct (**Figure 7A**). The *Eb*CHI overexpression vector *p*HB-*Eb*CHI-flag and the vector control *p*HB-flag were separately introduced into *E. breviscapus* to generate *Eb*CHI-OVX and CK hairy root cultures; meanwhile, hairy root lines generated through transformation with the blank C58C1 strain produced WT cultures.

PCR analyzes showed that these hairy root cultures contained *rolB*, the hygromycin resistance gene (*hyg*) and the exogenous *Eb*CHI gene in *Eb*CHI-OVX lines (**Figure 7C**). The *rolB* gene in the genome of *A. tumefaciens* indicated that the hairy roots had been induced by *A. tumefaciens*. The *hyg* gene indicated that the plasmid pHB-flag or *p*HB-*Eb*CHI-flag had been transformed into the hairy roots. The PCR product consisting of the *Eb*CHI 3′ fragment and vector fragment indicated that exogenous *Eb*CHI had been successfully introduced into the genome of the *E. breviscapus* hairy roots.

A qPCR analysis indicated that, in comparison with the levels of the WT control, the *Eb*CHI expression levels in all the five independent *Eb*CHI-OVX lines (OVX-1, 2, 3, 4, and 5) were significantly enhanced (3.7-, 9.6-, 3.8-, 7.4-, and 4.5-fold). No significant difference was observed between the WT and CK lines (**Figure 7B**). These results indicated that the exogenous *Eb*CHI gene had been successfully integrated and was highly expressed in *E. breviscapus* hairy root cultures.

Metabolite analysis showed that the contents of scutellarin in the *Eb*CHI-OVX lines were significantly higher than those in the WT and CK lines, and the scutellarin accumulations in different independent lines were closely correlated with the corresponding *Eb*CHI expression levels (**Figure 7B**). Line OVX-2, with the highest *Eb*CHI expression, produced the most abundant scutellarin (2.21 mg g^-1^, dw), which was ∼2.4- and 3.1-fold higher than that in its WT (0.90 mg g^-1^, dw) and CK (0.71 mg g^-1^, dw) counterparts, respectively.

## Discussion

Metabolic pathways are very complex. It is therefore difficult to exactly target the best intervention points to obtain the best outcome. However, biotechnological advances in data mining are taking away much of the guesswork by allowing the impact of modifications to be predicted more accurately (Saito and Matsuda). In particular, gene-metabolite network analysis is a powerful tool for decoding the functions of genes and screening rate-limiting enzymes (Cline et al., 2007). This knowledge-driven approach to engineering requires a well-characterized population as a source of genetic variation. The function of plant hormones, such as MeJA, to trigger secondary metabolism in cell cultures has made them powerful tools to examine genetic diversity and metabolic alterations and help to reveal the intricacies of the cellular process.

JA, including jasmonic acid and its oxylipin derivatives (such as MeJA, used in this study), is a powerful plant hormone that regulates many developmental processes and stress tolerance and resistance through secondary metabolism, including flavonoid biosynthesis (Hichri et al., 2011). The jasmonate ZIM-domain (JAZ) proteins are thought to repress JA-regulated responses through transcription factor interactions. In Arabidopsis, bHLH transcription factors (MYC2, 3 and 4) and R2R3 MYB transcription factors (MYB21 and 24) are direct targets of JAZ proteins (Marie and Meike, 2015). According to studies in other plants, we speculated that JAZ proteins interacted with the bHLH and R2R3 MYB of the WD-repeat/bHLH/MYB (MBW) complex in *E. breviscapus* and repressed JA-regulated flavonoid accumulation. Then, exogenous MeJA induced the degradation of JAZ proteins through the SCFCOI1-26S proteasome pathway, and the released MBW complexes activated early biosynthetic genes (EBGs) of the flavonoid pathway, including CHS, CHI, F3H, FNS, and F3′H (Xu et al., 2015).

To predict rate-limiting steps and key enzymes accurately, integrating transcript profiles and metabolic accumulations to establish a gene-metabolite network after disturbances (molecular signal, biotic and abiotic stress) has been achieved successfully in many pharmaceutically valuable metabolic pathways, such as the screening of AP2/ERF transcription factors and key enzymes involved in lignan biosynthesis in *I. indigotica* (Chen et al., 2015a), the characterization of steps in the rosmarinic acid biosynthetic pathways in *S. miltiorrhiza* (Xiao et al., 2010, 2015) and the isolation of regulators of aliphatic glucosinolate biosynthesis in *Arabidopsis* (Hirai et al., 2007). In this research, a combination of transcript and metabolite profiles of *E. breviscapus* after MeJA induction was used to draw a gene-metabolite network to determine the rate-limiting step, which indicated that *Eb*CHI, as a key enzyme affecting scutellarin biosynthesis in *E. breviscapus*, was the most closely related to scutellarin accumulation (**Figure 5**). In previous studies, a 12-fold higher level of apigenin was accumulated in CHI-overexpressing *Saussurea medusa* hairy roots than in WT hairy roots (Li et al., 2006). The overexpression of CHI could also increase the accumulation of flavonols in petunia and of flavone in *S. baicalensis* (Muir et al., 2001; Park et al., 2011). To validate the result of the network analysis, the function of *Eb*CHI was investigated both *in vitro* and *in vivo*. The overexpression of *Eb*CHI significantly increased scutellarin accumulation in *E. breviscapus* hairy roots compared with that in WT. These results demonstrated that *Eb*CHI plays an important role in scutellarin accumulation in *E. breviscapus*, consistent with the hypothesized regulatory roles.

Plants accumulate very rich and diverse bioactive metabolites, so the consumption of herbal medicines and medicinal plants is widespread and increasing. Because the main source of raw materials is from natural and wild fields, the problems of quality stability and overexploitation need to be addressed urgently. In recent years, significant progress has been made in the use of tissue and cell culture (Pistelli et al., 2010). With this tissue culture technology, genetic transformation is promising to modify pathways for the biosynthesis of bioactive compounds. Hairy roots are prominent in their genetic and biosynthetic stability, and their fast growth offers an additional advantage to their use as a continuous source of large quantities of valuable secondary metabolites, replacing natural sources (Ming et al., 2013). Here, we first established hairy root cultures of *E. breviscapus* under optimal conditions to produce the bioactive compound scutellarin. To produce a high concentration of scutellarin, *Eb*CHI-overexpressing hairy root cultures were established for metabolic engineering to enhance scutellarin accumulation. Compared with the 2.6 mg g^-1^ (dw) and 0.21 mg g^-1^ (dw) in natural whole plant and root, 2.21 mg g^-1^ (dw) was found in these transgenic hairy root cultures.

## Conclusion

In conclusion, the current study selects more suitable intervention points to engineer the production of target metabolites effectively through a knowledge-driven approach combining gene expression and metabolite accumulation data. The method of integrating transcript and metabolite data using MeJA-elicited *E. breviscapus* as a source of variation of gene expression and metabolites provided new insights into scutellarin biosynthetic genes. *Eb*CHI is undoubtedly a key rate-limiting step in the scutellarin biosynthetic pathway. *Eb*CHI-overexpressing hairy root cultures accumulated high levels of scutellarin, providing a promising method to relieve the overexploitation and shortage of *E. breviscapus* in the future.

## Author Contributions

RC, TZ, JL, LZ, and WC conceived and designed the study. RC, TZ, and JY performed the experiments. HT conducted the experiment of subcellular localization. RC, JL, and QL contributed to data analysis and bioinformatics analysis. RC, TZ, and SG analyzed the accumulation of compounds through HPLC-MS/MS and HPLC-UV. RC, TZ, and JL wrote the final manuscript. All authors read and approved the final version of the manuscript.

## Conflict of Interest Statement

The authors declare that the research was conducted in the absence of any commercial or financial relationships that could be construed as a potential conflict of interest.

## Footnotes

1 http://www.megasoftware.net/

2 https://www.ncbi.nlm.nih.gov/

3 https://npsa-prabi.ibcp.fr/NPSA/npsa_sopma.html

4 http://www.sbg.bio.ic.ac.uk/phyre2/html/page.cgi?id=index

5 http://www.sbg.bio.ic.ac.uk/phyre2/html/page.cgi?id=index

## Supplementary Material

FIGURE S1 Mass spectra, product ion spectra and representative MRM chromatograms of five compounds. **(A)** Optimized MRM parameters for five metabolites. **(B)** Mass spectrum and product ion spectrum of the five compounds. **(C)** Representative MRM chromatograms of five compounds.

FIGURE S2 Characterization, phylogenetic analysis and subcellular localization of the *Eb*CHI protein. **(A)** A phylogenetic tree of the CHI proteins from various species. **(B)** Comparison of the predicted amino acid sequence of *Eb*CHI with those of related proteins. The *Eb*CHI sequence was aligned with *Cm*CHI (AEP37358.1), *Ct*CHI (ALG75881.1), *At*CHI (AEE79342.1), *Nt*CHI (NP_001312216.1), *Zm*CHI (NP_001149585.1), and *Ls*CHI (BAS69315.1). **(C)** The domain of *Eb*CHI. **(D)** Secondary structure of the *Eb*CHI protein. **(E)** 3-D structure of the *Eb*CHI protein. **(F)** Subcellular localization of *Eb*CHI. **a**, A rice protoplast expressing *Eb*CHI-GFP showing green fluorescent signal; **b**, the same protoplast cell showing chlorophyll autofluorescence in the plastids; **c**, merge of panels **a,b**; **d**, bright-field image; **e**, a rice protoplast expressing GFP showing green fluorescent signal; **f**, the same protoplast cell showing chlorophyll autofluorescence in the plastids; **g**, merge of panels **e,f**; **h**, bright-field image. Bars = 5 nm.

FIGURE S3 Hairy root cultures. **(A)** Explants. **(B)** and **(C)** Induced hairy roots.

TABLE S1 Primers used in this experiment.

TABLE S2 Contents of five flavone metabolites.

TABLE S3 Canonical correlation analysis.

TABLE S4 Nucleotide and protein sequences of *Eb*CHI.

TABLE S5 Different transformation conditions of hairy root culture.
