# Supplementary figures and images for: Integrated Transcript and Metabolite Profiles Reveal That EbCHI Plays an Important Role in Scutellarin Accumulation in Erigeron breviscapus Hairy Roots
Source: Front Plant Sci. 2018 Jun 21;9:789. doi: 10.3389/fpls.2018.00789 (PMC6036287; doi:10.3389/fpls.2018.00789)

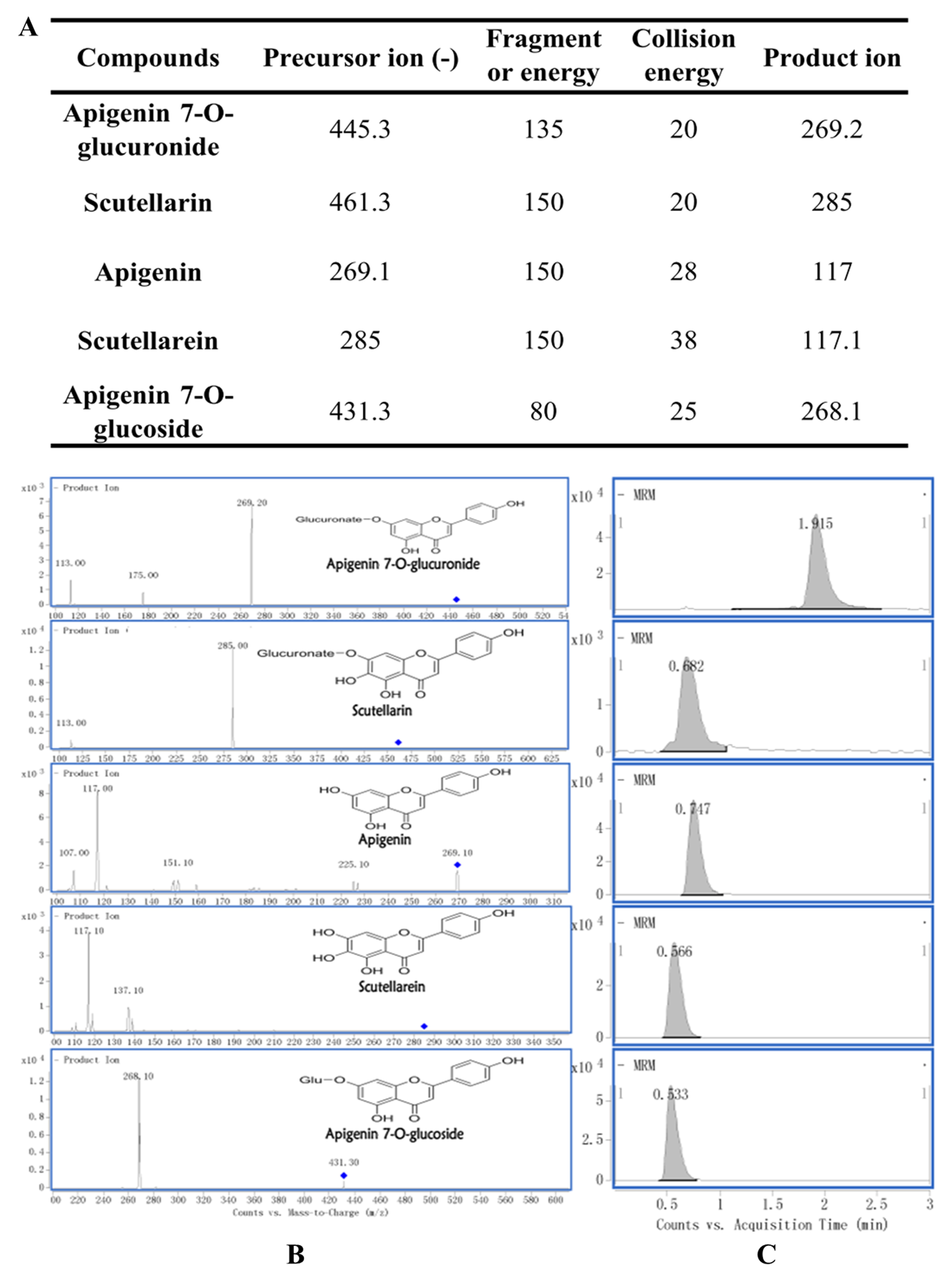

Supplement: FIGURE S1 — Mass spectra, product ion spectra and representative MRM chromatograms of five compounds. (A) Optimized MRM parameters for five metabolites. (B) Mass spectrum and product ion spectrum of the five compounds. (C) Representative MRM chromatograms of five compounds. [file Image_1.JPEG]

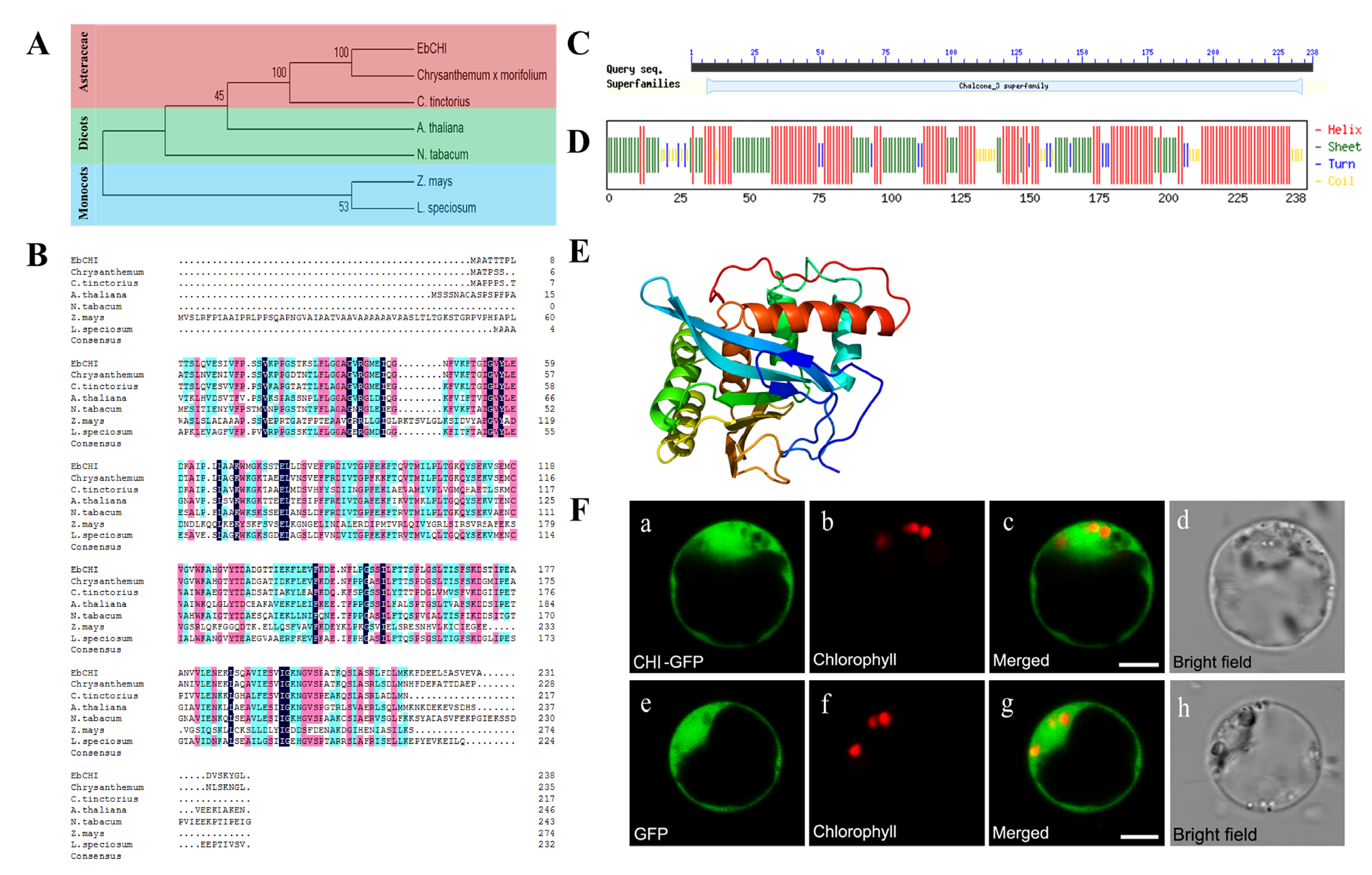

Supplement: FIGURE S2 — Characterization, phylogenetic analysis and subcellular localization of the EbCHI protein. (A) A phylogenetic tree of the CHI proteins from various species. (B) Comparison of the predicted amino acid sequence of EbCHI with those of related proteins. The EbCHI sequence was aligned with CmCHI (AEP37358.1), CtCHI (ALG75881.1), AtCHI (AEE79342.1), NtCHI (NP_001312216.1), ZmCHI (NP_001149585.1), and LsCHI (BAS69315.1). (C) The domain of EbCHI. (D) Secondary structure of the EbCHI protein. (E) 3-D structure of the EbCHI protein. (F) Subcellular localization of EbCHI. a, A rice protoplast expressing EbCHI-GFP showing green fluorescent signal; b, the same protoplast cell showing chlorophyll autofluorescence in the plastids; c, merge of panels a,b; d, bright-field image; e, a rice protoplast expressing GFP showing green fluorescent signal; f, the same protoplast cell showing chlorophyll autofluorescence in the plastids; g, merge of panels e,f; h, bright-field image. Bars = 5 nm. [file Image_2.JPEG]

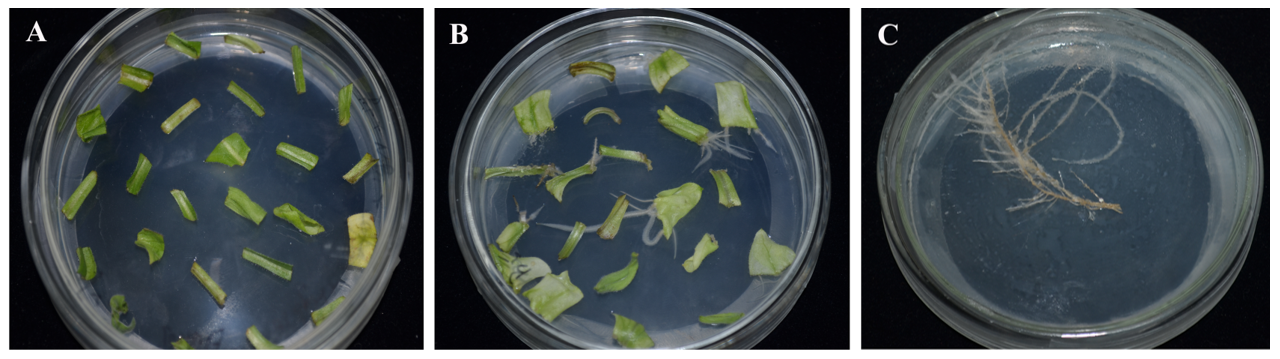

Supplement: FIGURE S3 — Hairy root cultures. (A) Explants. (B) and (C) Induced hairy roots. [file Image_3.PNG]
