# Supplementary material for: Integrated Transcript and Metabolite Profiles Reveal That EbCHI Plays an Important Role in Scutellarin Accumulation in Erigeron breviscapus Hairy Roots
Source: Front Plant Sci. 2018 Jun 21;9:789. doi: 10.3389/fpls.2018.00789 (PMC6036287; doi:10.3389/fpls.2018.00789)
Supplement: TABLE S1 — Primers used in this experiment. [file Table_1.PDF]

S1 Primers used in this study

primers for qRT-PCR

Name	Sequences 5’-3’	Tm (℃)
qCHI-F	TCCGGGACATTGTTACAGGC	62
qCHI-R	GATGAGCCTGGCAGGAAGTT	62
qFNS-F	ATCATCGCCACTGCCCTTTT	60
qFNS-R	CGAGCCAAGACGGAGATGAA	62
qCHS-F	GTCCCAAAGCTCGGTCAAGA	62
qCHS-R	CATGCCGCCTGCATAACATC	62
qF3H-F	TCGATATGACCGGTGGCAAG	62
qF3H-R	CAAGCCAGGCCCATTAACAC	61
UBC-F	ACCCTCACGGGGAAGACCATC	62
UBC-R	ACCACGGAGACGGAGGACAAG	62

primers for RT-PCR

Name	Sequences 5’-3’	Tm (℃)
CHI-F	ATGCCTGCCACGACA	61
CHI-R	TTACAAACCATATTTGCTTACATC	59
*p*ET-32a-*Eb*CHI-F	GATCGGATCCATGCCTGCCACGACAACACCATTA	61
pET-32a-*Eb*CHI-R	GATCGAATTCTTACAAACCATATTTGCTTACATC	59
*p*HB-*Eb*CHS-flag-F	GAGGATCCATGGTAAGAAAGCGATA	68
*p*HB-*Eb*CHS-flag-R	ATTAACTTCGGTCATTAGAGGC	60
*p*1301-*Eb*CHI-GFP-F	AACCATGGCTGCCACGACAACACCAT	68
*p*1301-*Eb*CHI-GFP-R	GCAGATCTCAAACCATATTTGCTTAC	66

Primers for hairy roots detection

Name	Sequences 5’-3’	Tm (℃)
*p*HB-*Eb*CHI-1F	GGATTCTGTTGAGTTCTTCC	65
Rbcs-R	ATTAACTTCGGTCATTAGAGGC	62
rolB-F	GCTCTTGCAGTGCTAGATTT	60
RolB-R	GAAGGTGCAAGCTACCTCTC	60
hyg-F	CGATTTGTGTACGCCCG	62
hyg-R	CGATGTAGGAGGGCGTG	63
