# Supplementary material for: Integrated Transcript and Metabolite Profiles Reveal That EbCHI Plays an Important Role in Scutellarin Accumulation in Erigeron breviscapus Hairy Roots
Source: Front Plant Sci. 2018 Jun 21;9:789. doi: 10.3389/fpls.2018.00789 (PMC6036287; doi:10.3389/fpls.2018.00789)
Supplement: TABLE S2 — Contents of five flavone metabolites. [file Table_2.PDF]

S2 Details of contents of five compounds

			Content %				
			Apigenin-7-O-glucuronide	Scutellarin	Apigenin	Scutellarein	Apigenin-7-O-glucoside
1d	Control	Mean	0.0235	0.2661	0.0008	0.0024	0.0017
		SD	0.0020	0.0092	0.0000	0.0002	0.0007
	MeJA treatment	Mean	0.0269	0.2580	0.0009	0.0023	0.0016
		SD	0.0023	0.0321	0.0001	0.0003	0.0002
2d	Control	Mean	0.0208	0.2372	0.0005	0.0020	0.0007
		SD	0.0022	0.0376	0.0001	0.0003	0.0000
	MeJA treatment	Mean	0.0359	0.4087	0.0035	0.0027	0.0015
		SD	0.0039	0.0191	0.0005	0.0004	0.0003
3d	Control	Mean	0.0271	0.2579	0.0005	0.0019	0.0007
		SD	0.0011	0.0306	0.0001	0.0002	0.0003
	MeJA treatment	Mean	0.0349	0.4658	0.0019	0.0040	0.0041
		SD	0.0025	0.0129	0.0002	0.0003	0.0005
4d	Control	Mean	0.0298	0.4536	0.0006	0.0028	0.0016
		SD	0.0055	0.1734	0.0000	0.0012	0.0004
	MeJA treatment	Mean	0.0256	0.2947	0.0012	0.0029	0.0024
		SD	0.0040	0.0330	0.0001	0.0004	0.0003
5d	Control	Mean	0.0208	0.3252	0.0007	0.0016	0.0015
		SD	0.0014	0.0209	0.0002	0.0002	0.0005
	MeJA treatment	Mean	0.0210	0.2932	0.0009	0.0013	0.0013
		SD	0.0018	0.0249	0.0001	0.0002	0.0002
6d	Control	Mean	0.0190	0.2609	0.0006	0.0017	0.0007
		SD	0.0008	0.0305	0.0002	0.0002	0.0003
	MeJA treatment	Mean	0.0257	0.2617	0.0006	0.0021	0.0009
		SD	0.0016	0.0851	0.0001	0.0003	0.0002
7d	Control	Mean	0.0198	0.3112	0.0006	0.0024	0.0013
		SD	0.0198	0.3112	0.0006	0.0024	0.0013
	MeJA treatment	Mean	0.0235	0.3097	0.0007	0.0022	0.0012
		SD	0.0010	0.0257	0.0002	0.0002	0.0000
8d	Control	Mean	0.0245	0.3330	0.0006	0.0018	0.0014
		SD	0.0057	0.0338	0.0001	0.0001	0.0001
	MeJA treatment	Mean	0.0303	0.3056	0.0010	0.0023	0.0015
		SD	0.0097	0.0411	0.0002	0.0004	0.0001
