# Supplementary material for: Integrated Transcript and Metabolite Profiles Reveal That EbCHI Plays an Important Role in Scutellarin Accumulation in Erigeron breviscapus Hairy Roots
Source: Front Plant Sci. 2018 Jun 21;9:789. doi: 10.3389/fpls.2018.00789 (PMC6036287; doi:10.3389/fpls.2018.00789)
Supplement: TABLE S3 — Canonical correlation analysis. [file Table_3.PDF]

S3 canonical correlation analysis

correlation variables between genes and metabolites

	Apigenin	Apigenin 7-O-glucoside	Apigenin 7-O-glucuronide	Scutellarein	Scutellarin
CHI	0.895161	0.104695	0.719105	-0.04064	0.642144
CHS	0.890584	0.320717	0.858888	0.182944	0.795143
F3H	0.478821	0.18417	0.347396	0.308098	0.366921
FNS	-0.10956	0.140314	-0.23944	0.501047	-0.19698

canonical correlation analysis

canonical correlation variables (between U and V)	0.916

correlation variables between genes and U	
CHI	0.86
CHS	0.949
F3H	0.363
FSN	0.325

correlation variables between compounds and V	
Apigenin-7-glucuronide	0.279
Scutellarin	0.199
Apigenin	-0.4
Scutellarein	0.714
Apigenin-7-glucoside	0.695
