# Supplementary material for: Integrated Transcript and Metabolite Profiles Reveal That EbCHI Plays an Important Role in Scutellarin Accumulation in Erigeron breviscapus Hairy Roots
Source: Front Plant Sci. 2018 Jun 21;9:789. doi: 10.3389/fpls.2018.00789 (PMC6036287; doi:10.3389/fpls.2018.00789)
Supplement: TABLE S4 — Nucleotide and protein sequences of EbCHI. [file Table_4.PDF]

S4 Sequences of *Eb*CHI

>*EbCHI*
ATGGCTGCCACGACAACACCATTAACCACCAGTCTCCAAGTTGAATCCATCGTTTTTCC
GTCGTCTGTCAAGCCTCCCGGCTCGACCAAATCTTTGTTCCTCGGTGGCGCTGGTGTG
AGAGGTATGGAAATCCAAGGGAACTTTGTGAAGTTTACGGGAATTGGTGTGTATTTAG
AGGATAAAGCTATTCCATTACTCGCTGCTAAGTGGATGGGCAAATCTTCTACTGAGTTG
CTGGATTCTGTTGAGTTCTTCCGGGACATTGTTACAGGCCCCTTTGAAAAATTTACTCA
GGTGACAATGATACTTCCATTAACTGGTAAACAATACTCTGAGAAAGTATCGGAAATGT
GTGTCGGTGTTTGGAAAGCTCATGGGGTCTATACAGACGCAGATGGCACAACCATTGA
AAAGTTTCTTGAGGTTTTCAAGGATGAAAACTTCCTGCCAGGCTCATCTATTCTCTTTA
CAACTTCGCCTCTTGGATCACTGACGATCAGCTTCTCGAAAGATAGTACCATACCTGAA
GCTGCGAATGTAGTATTAGAGAATGAAAAATTATCACAAGCAGTGATTGAGTCAGTGAT
TGGGAAGAATGGTGTTTCCCCAGCAACCAAACAAAGCTTGGCATCAAGACTCTTCGAC
CTCATGAAAAAGTTTGATGAGGAATTAAGTGCAAGTGTCGAAGTGGCAGATGTAAGCA
AATATGGTTTGTAA

>EbCHI-Protein
MAATTTPLTTSLQVESIVFPSSVKPPGSTKSLFLGGAGVRGMEIQGNFVKFTGIGVYLEDK
AIPLLAAKWMGKSSTELLDSVEFFRDIVTGPFEKFTQVTMILPLTGKQYSEKVSEMCVGV
WKAHGVYTDADGTTIEKFLEVFKDENFLPGSSILFTTSPLGSLTISFSKDSTIPEAANVVLE
NEKLSQAVIESVIGKNGVSPATKQSLASRLFDLMKKFDEELSASVEVADVSKYGL
