# Supplementary material for: Integrated Transcript and Metabolite Profiles Reveal That EbCHI Plays an Important Role in Scutellarin Accumulation in Erigeron breviscapus Hairy Roots
Source: Front Plant Sci. 2018 Jun 21;9:789. doi: 10.3389/fpls.2018.00789 (PMC6036287; doi:10.3389/fpls.2018.00789)
Supplement: TABLE S5 — Different transformation conditions of hairy root culture. [file Table_5.PDF]

S5 Culture conditions of hairy root culture

Transformation conditions			Transformation rate (%)
OD_{600} (*A. tumefaciens*)	Induction time (Minutes)	Co-culture time (Days)	
0.4	5	0	10
		1	14
		2	10
		3	12
	10	0	14
		1	16
		2	26
		3	28
	15	0	16
		1	18
		2	32
		3	30
**0.6**	5	0	14
		1	18
		2	22
		3	30
	**10**	0	18
		1	34
		**2**	**60**
		3	46
	15	0	20
		1	42
		2	34
		3	18
0.8	5	0	22
		1	32
		2	38
		3	36
	10	0	26
		1	58
		2	26
		3	10
	15	0	8
		1	0
		2	0
		3	0
